# Scale of Assessment of Caregiver Care Burden of People With Dementia: A Systematic Review of Literature

**DOI:** 10.37825/2239-9747.1044

**Published:** 2023-12-29

**Authors:** Veronica Strini, Angela Prendin, Valentina Cerrone, Roberta Schiavolin, Ilaria de Barbieri, Vincenzo Andretta, Luigi Fortino, Biagio Santella, Gianluigi Franci, Mario Capunzo, Giovanni Boccia

**Affiliations:** a Clinical Research Unit, University-Hospital of Padua, Padua, Italy; b Palliative Care and Antalgic Therapy/Pediatric Hospice, University-Hospital of Padua, Padua, Italy; c A.O.U. San Giovanni di Dio e Ruggi D’Aragona, U.O.C. Oncology, 84131 Salerno, Italy; d Continuity of Care Service-University-Hospital of Padua, Padua, Italy; e Department of Health Professions, University Hospital of Padua, Padua, Italy; f A.O.U. San Giovanni di Dio e Ruggi D’Aragona, U.O.C. Hospital Hygiene and Epidemiology, 84131 Salerno, Italy; g Department of Medicine, Surgery and Dentistry, University of Salerno, 84081 Salerno, Italy

**Keywords:** Burden, Caregiver, Nurse, Dementia

## Abstract

Cases of dementia have increased significantly in recent years. The family represents the main cornerstone of assistance to the elderly suffering from dementia, in particular the caregiver. Family members who take on the role of caregiver are subjected to physical, psychological, emotional, social and financial stress, which can be conceptualised with the term “burden”. The aim of this study was to investigate which tools are best suited to measure the type of burden based on the type of dementia for each caregiver.

A literature search was undertaken in MEDLINE, CINHAL and The Cochrane Database in September 2022, including articles from the last 20 years and using a combination of keywords and defined inclusion criteria. This literature review has been performed according to the PRISMA statement.

From a total of 116 articles regarding the use of burden rating scales for caregivers, 18 scales were selected.

The review provides a useful overview of burden assessment scales, classified into three categories, one-dimensional, multidimensional, or distinct concept with a subjective and objective component, in order to adopt appropriate strategies to assess caregiver burden and improve the quality of their health, both in the community and in hospitals. Indeed, the domestic context is the most studied as there is a greater risk of developing the burden of the caregiver: for this reason, some scales include the assessment of both the caregiver and the patient receiving treatment.

## 1. Introduction

In the population, there is a constant increase in the number of older people thanks to the progress of medicine and therefore to the increase in life expectancy [1]. Among the various consequences of this important socio-demographic transformation is the increasing incidence of chronic degenerative diseases such as dementia, considered the leading cause of disability in old age [2]. Symptoms of dementia, such as primarily a decrease in memory, orientation, and language skills, are imperceptible at first and slowly progress to the point where they appear evident and devastating. These symptoms are classified into three categories and concern the cognitive, functional, and behavioural spheres and destroy people’s ability to operate [3]. The most common forms of dementia are Alzheimer’s (50–60 %) and vascular dementia (10–20 %), while less frequent dementias (15 %) include Parkinson’s disease, immunodeficiency-related dementia (AIDS), and Pick’s disease [4]. Alzheimer’s disease is a long-lasting, degenerative, and irreversible disease. The patient suffering from Alzheimer’s disease progressively loses the ability to carry out normal daily activities. This results in the need for one or more individuals to take care of him [5]. In the scenario of helping people with dementia, the family represents the fundamental cornerstone of care [6]. The caregiver perceives the relationship with the sick person, care work, physical tiredness, social isolation, lack of family support, lack of support from services and financial limitations as stressful factors [7]. Effects may include increased levels of depression and anxiety, increased use of psychiatric drugs, a decline in physical health, impaired immune function, increased mortality [8], sleep-wake rhythm disturbances, irritability, and disturbances of sleep [9]. The long duration of the disease, the Early loss of self-sufficiency and the need for continuous care are therefore a heavy and long-term commitment for the family [10].

In 2017, the WHO launched a global action plan on the public health response to dementia for 2017–2025 [11]. To support dementia risk reduction in different countries, the WHO published guidelines on risk reduction in cognitive decline and dementia [12]. The guidelines highlight that many of the modifiable risk factors for dementia are shared with other noncommunicable diseases (NCDs).

A systematic review reported that the role of the nurse appears to be fundamental to decreasing behavioural and psychological symptoms of dementia and caregiver stress with dementia care management [13]. The caregiver experiences high levels of emotional and psychological tension until becoming a second victim of the disabling diseases that affect older people [14]. The role of the nurse in a context of fragmentation of care due to high specialties can involve preventive investigations of alarm signals about the well-being of the patient and his family at an early stage [15,16].

It is noteworthy that the time spent providing care was considered an important factor in caregiver burden, as this was not strongly emphasised in the previous systematic reviews [17,18]. Wang et al. study showed that Chinese caregivers spent an average of 127.6 h per week on care, while female caregivers performed caregiving tasks 2.5 times more than male caregivers [3,19]. Some studies have reported that caregivers are significantly more vulnerable to suffering from anxiety and depression [20]. The psychological burden has become the most important aspect [21], and this may be related to the fact that the caregiver’s life is mainly focused on caring for the patient and has no time for personal life, employment, or social life, which creates a strong role conflict [20].

It becomes essential, especially for caregivers, to determine the level, type and cause of burden for people with dementia.

The results of the survey can be conveyed through the use of appropriate measurement tools. To investigate which tools are best suited to measure the type of burden, according to the type of subject, of each caregiver, it was decided to carry out a systematic review of the literature.

Objective to investigate which tools are best suited to measure the type of burden, according to the type of subject of each caregiver.

## 2. Methods

In September 2022, a comprehensive literature search was conducted using MEDLINE, The Cochrane Database, and CINHAL (consulted on September 7, CINHAL on September 15, and the Cochrane Database on September 18). The search included articles published in the last ten years. The PEO methodology was used for the literature search:

P: caregivers of subjects with dementia;

E: the care load on caregivers;

O: early identification of subjects at risk in clinical practise;

The search strategy used MeSH terms and keywords to identify the potential studies. Search terms chosen were “dementia” OR “vascular dementia” OR “senile dementia” OR “mixed dementia” AND “caregiver” OR “caregiv*” OR “carer*” OR “family caregiver” OR “informal caregiver” OR “home caregiver” AND “burden” OR “strain” OR “stress” OR “distress” OR “suffer” OR “overload”.

The inclusion of the selected articles complies with the following inclusion and exclusion criteria:

Inclusion criteria: articles that focus on the creation, validation, criticism and use of tools for assessing the level of care burden of caregivers of individuals with dementia.

Exclusion criteria: articles with evaluation scales of caregiver burden of demented subjects in the terminal phase are excluded since the assistance could suffer bias due to other care problems related to the patient’s pathology. The studies investigated that included the caregivers under the age of 18, were excluded.

This literature review has been performed according to the PRISMA statement. The quality assessment of the studies included in the systematic review has been performed using CASP checklist. To optimise the search, the following limits have been applied: articles presenting abstracts and publications written in English and/or Italian. The articles that have relevance in the title and abstract to the clinical question were selected. Two authors independently selected the articles of greatest interest, which met the search criteria. Furthermore, a third reviewer subsequently compared and confirmed the selected studies or requested modifications. The quality assessment of the studies included in the systematic review has been performed using CASP checklist.

## 3. Results

**Fig. 1:**
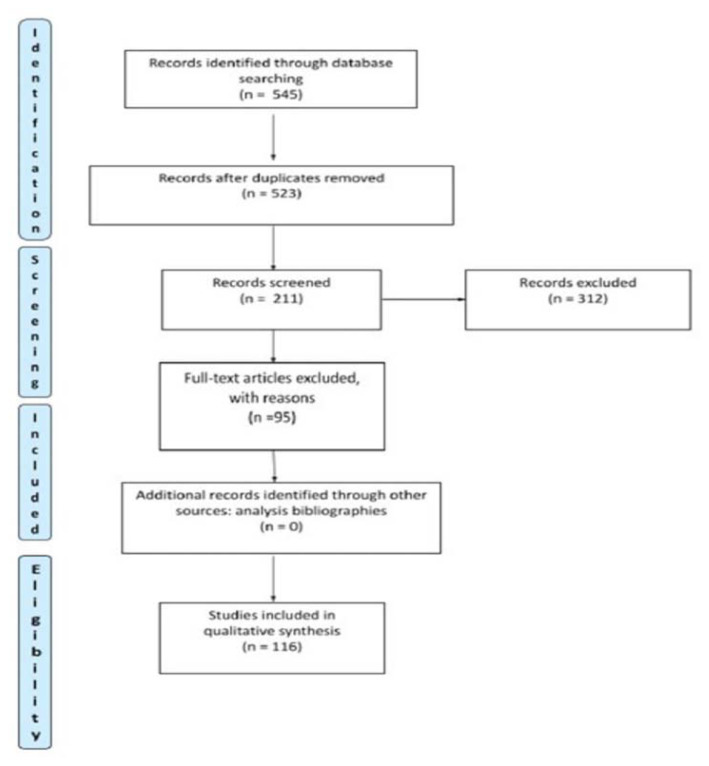
Preferred Reporting Items for Systematic Reviews and Meta-Analyses Guideline.

545 articles were identified. A total of 336 articles were eliminated, of which 22 were duplicates, as they concerned aspects not relevant to our review. Respecting the inclusion and exclusion criteria, 95 articles were finally eliminated from the full text analysis of 211 articles. These are studies that present rating scales that investigate the load in a marginal and non-exclusive way. In these articles, care load is not the main dimension to be investigated, and scales only investigate factors linked to the concept of load (strain, stress or anxiety). Furthermore, articles in which the scale is used for the validation of other assessment tools not related to the concept of burden are not included. A tool that assessed the needs of the family was excluded. Fig. 1 shows the research phases. Consequently, 116 articles are included in the review study that focus on the creation, validation, use and critique of burden rating scales for caregivers of patients with dementia (Fig. 1).

## 4. Discussion

The Zarit Burden Interview (ZBI) is a self–report scale aimed at assessing the subjective burden experienced by a caregiver [22,23]. It consists of 22 questions relating to problems that arise in different areas: health and well-being, personal and social life and finances. The administration time is approximately 10 min. The Zarit scale has also been used in multiple studies specifically targeting caregivers of patients with Parkinson’s disease, whose neuropsychiatric symptoms (NPS) can be particularly burdensome for caregivers and whose impact can be effectively assessed through the ZBI [24]. It is considered the most commonly used measure of caregivers’ burden. This widespread use has prompted questions about the comparability of results internationally. Therefore, research into the validity of the ZBI across countries and cultures is crucial for the quality of epidemiological, clinical and health services research, as well as for routine clinical activities. ZBI has been translated into 18 languages [25–27]. There are numerous research studies that contemplate the ZBI: to determine the association between care weight and depression in caregivers who care for subjects with dementia; to examine the characteristics of family caregivers and assess whether income, subjective health, age, and relationship are associated with the burden of care they experience to identify the impact of variables such as sex, length of time, style of care, depression, and the perception of the burden of caregiving on the physical and psychological well-being of the caregivers of people with dementia [28,29].

The Caregiver Burden Scale (CBS) was also engineered to be a valid tool for diagnosing the subjective burden of caregivers, regardless of the diagnosis of the person receiving the care; it was used to measure the care burden. It aims at people with various diagnoses such as stroke, dementia, haemophilia, Parkinson’s disease, heart failure, traumatic brain injury, and long-term disease, disability and/or old age [24,30]. It consists of 22 items divided into five domains. The administration time is approximately 10–15 min. In 1999, the reliability and validity of the Chinese version of CBS were established for the assessment of Chinese caregivers of patients with Alzheimer’s dementia [31]. It has been widely used to study the factors determining a positive (satisfaction) and negative (burden) evaluation among American caregivers of patients with Alzheimer’s disease, or, in conjunction with other scales, to determine factors associated with the satisfaction of family members caring for patients with dementia in the home setting [32,33].

The Burden Scale for Family Caregivers (BSFC) was designed for caregivers who provide home care to family members with neurological disorders such as dementia and stroke. It is a simple tool consisting of 28 questions grouped in five dimensions. It is designed for use in both clinical and research settings [34]. It takes approximately 5–10 min to complete. BSFC has been validated in large samples and in several languages (e.g., Turkish and Danish) [35,36].

Graessel, Berth, Lichte and Grau developed and validated a quick and inexpensive way to assess subjective burden, a short form of the 10-item BSFC (BSFC-s), each representative of a description of the possible effects of caregiver burden classified on a 4-point scale. The administration time is 10 min [37,38].

The Self-Rated Burden Scale (SRB) is a care burden self-assessment tool consisting of a single visual analogue scale ranging from 0 to 100, where a high score indicates a high level of burden [39]. The administration time is approximately 5 min. SRB has been shown to be a valid measure of subjective caregiver burden and is recommended for use in clinical practise, with particular utility for rapid screening of at-risk caregivers. However, the scale requires further investigation in the event of a high score and for this reason, it is often associated with the Caregiver Strain Index (CSI) to complete the load assessment [40].

The Relative Stress Scale (RSS) was also developed to measure the perceived burden of caregivers caring for individuals with dementia [41]. The RSS consists of 15 distinct questions. Higher scores reflect a greater perceived burden on the caregiver. The RSS items can be divided into three subgroups: emotional distress, social distress and negative feelings [42]. The administration time is approximately 10–20 min. RSS can be used to identify the characteristics of the caregiver and the patient associated with various aspects of the burden, guiding the identification of targeted interventions [43]. In the clinical setting, it can also be used to measure the effectiveness of an intervention and detect its effects on caregivers and patients [42].

Similarly, the Self-Perceived Pressure from Informal Care Scale (SPPIC) was specially developed to map the caregiver’s experience. The SPPIC is a 9-item scale. The administration time takes more than 10 min. Perceived pressure refers to the needs of the care situation in proportion to the personal interests of the caregiver. These interests refer to the caregiver’s need for non-care-related thoughts, activities and roles [44].

These six scales measure the perceived burden of caregivers. In particular Zarit, in the formulation of the ZBI scale, treated the burden as a one-dimensional variable [22]. Subsequent studies found that evaluating the load as a one-dimensional variable was insufficient and that the tools had to separate the objective and subjective aspects of the load [45].

Montgomery et al. attempted to specifically delineate the objective and subjective burdens in the Montgomery-Borgotta Caregiving Burden Scale questionnaire [46]. This scale, in fact, differentiates between subjective and objective components of the load that gave rise to the concepts of subjective burden and objective burden and points to the multidimensionality of the impact of the treatment. It consists of three relatively independent main variables that aim to cover most of the variance of the concept (violation of aspects of privacy, perception of responsibility for care and the emotional impact of the latter) [46]. The administration time takes less than 20 min. The scale was used on a sample of caregivers of patients with dementia in a study aimed at exploring the relationship between communication problems associated with dementia and the burden of the caregiver in the context of problematic behaviours and the cognitive and functional abilities of the recipient [47].

Another scale that evaluates the two-dimensionality of the burden is the Family Burden Interview Schedule (FBIS). Its primary purpose was to measure the amount of stress that family members of people with schizophrenia patients in the home environment. This scale is composed of 24 items that evaluate the objective burden of care and 1 item that evaluates the subjective burden of the caregiver and family members involved in the care. The items for the assessment of the burden of care were grouped into six domains and presented in the form of a question to recreate the interview form. Administration time takes less than 20 min. The tool can be used in a variety of studies, for example, to compare different treatment situations for similar diseases or to compare the effects on families of dementia patients [48].

The Screening of Caregiver Burden (SCB) was performed to quickly identify the load of care in caregiver activities. The tool provides information on two different burden areas: objective and subjective. This is a measure created for caregivers of patients suffering from Alzheimer’s disease through the application of 25 items. Objective burden implies the number of potentially negative experiences of the caregiver, whereas subjective burden refers to the extent of suffering experienced in response to caregiving experiences [49]. A 2005 study provided a shorter version of SCB that healthcare professionals can use to quickly assess the burden associated with care. The 7-item SCB was proposed after a demonstration of good internal consistency. These seven questions are simple and allow you to obtain the caregiver’s profile, including social, emotional and physical aspects, as well as an assessment of the patient’s status. This rating scale could easily be incorporated into a standard form that healthcare providers could administer in the waiting room, efficiently, within minutes, or by telephone during follow-up [50]. The administration time is less than 30 min.

The Burden Assessment Scale (BAS) was developed to assess the family burden related to the care of individuals with severe mental disorders. BAS is a 19-item scale that focuses on the objective burden and subjective consequences of the caregiver. Ten elements assess the objective burden and refer to observable behavioural effects caused by caregiving; nine elements evaluate the subjective aspects of the care burden. It appears as a 4-point Likert scale with 19 covered areas. The administration time is 15 min. Two independent studies that used the BAS scale reported improved reliability and sensitivity of the measure over time when it was self-administered. Finally, the authors underline, in addition to the short administration time compared to other tests, its use as a reliable tool for evaluating the effectiveness of an intervention on the quality of caregiver assistance [51]. The Burden Assessment Schedule is designed to assess the caregiver burden of subjects with chronic mental illness. The tool evaluates both the objective and subjective domains of the caregiver’s burden. The 40 items are grouped on the basis of nine domains [52]. The administration time takes about 30 min. A modified version of 20 items is also proposed for use in the definition of the caregiver care burden of patients with Parkinson’s disease [53]. The items on the scale are grouped into 5 domains (4 items for each domain) [52].

These scales do not distinguish between subjective and objective factors when evaluating the multidimensional aspect of the burden.

The Dementia Burden Scale-Caregiver is a scale that evaluates the multidimensional aspect on three fronts of the burden inherent in the caregiving activity aimed at patients with dementia [54]. The tool evaluates the fatigue resulting from care, the stress caused by the behavioural symptoms of the patient with dementia and the depressive symptoms experienced by caregivers through three existing scales.

The Modified Caregiver Strain Index (MCSI; 13 items), developed in 2003, is the most recent version of the Caregiver Strain Index designed in 1983. MCSI is a short, self-administered tool consisting of 13 questions that measure fatigue (“strain”) related to the assistance activity. It contains at least one element for each of the following main domains: financial, physical, psychological, social and personal.

The Neuropsychiatric Inventory-Questionnaire (NPI-Q) Distress Scale is a clinical tool for evaluating behavioural and psychological symptoms in dementia. It is based on an interview with the primary caregiver who must answer each of the 12 questions relating to 12 symptoms. The NPI-Q Distress Scale then provides information on the severity of symptoms and the level of distress perceived by the caregiver for each reported symptom, and, by adding up the scores for each domain, a total value is obtained regarding the severity of dementia and the level of global load perceived by the caregiver [8]. Finally, the caregiver’s depressive symptoms were measured using the nine-item version of the Patient Health Questionnaire (PHQ-9) developed for diagnosis, monitoring and determination of the severity of depression. PHQ-9 investigates the presence “in the last two weeks” of the nine symptoms of depression according to the DSM-IV [55].

The Caregiver Burden Inventory (CBI) was developed to measure the impact of caregiving on family members of patients, in particular those with Alzheimer’s disease and other forms of dementia [56]. The scale is developed by considering the burden as the result of multiple aspects of the caregiver’s life. The CBI allows the evaluation of various factors relating to the care load: objective load, psychological load, physical load, social load and emotional load. Scores for each item are assigned using a 5-point Likert scale [57]. The administration time is approximately 10–20 min. The result of the questionnaire provides a profile of the caregiver’s load in different areas, based on variations over time. The different profiles respond to the different social and psychological needs of caregivers useful for building targeted psychosocial interventions [58]. It has had applications to examine the emotional and sexual dimensions in partners involved as caregivers of patients with Alzheimer’s and to analyse the presence and relationship of specific socio-demographic variables, subjective burden and depressive symptoms among caregivers of patients with dementia [59,60].

The Caregiver Assessment of Difficulties Index (CADI) consists of 30 elements identified from the theoretical and empirical literature on care, which intend to represent aspects of social life, the economic situation, the relationship with the dependent family, professional and family support, addiction factors and the response to care based on caregiving needs (8 domains) [61,62]. In a study focusing on the psychometric properties of the scale used by caregivers of patients with dementia, the CADI was found to be an adequate tool for assessing the care burden of caregivers of people with dementia. The administration time was less than 20 min. The tool can also be used to measure changes in burdens over time, both short-term (to evaluate the impact of the intervention) and long-term (to monitor the different needs of the caregiver) [63]. In 2002, the Portuguese version of the Caregiver Difficulties Assessment Index was developed [64].

Finally, from the literature review, specific scales emerged for certain populations with minor use.

The Impact of Alzheimer’s Disease on the Caregiver Questionnaire (IADCQ) was developed in response to the lack of validated tools to measure the burden of Alzheimer’s disease on the caregiver. It is a 12-item instrument that measures the emotional, physical, social, financial, sleep, and time burdens associated with being a caregiver of Alzheimer’s patients. IADCQ is a useful questionnaire in clinical trials because it is self-administered, short and simple to evaluate [65].

The Berlin Inventory of Caregiver Burden of Patients with Dementia, originally in German (Berliner Inventar zur Angehörigenbelastung Demenz, BIZA-D), was developed to assess the objective and subjective burden of caregivers of patients with dementia. It consists of 88 items regarding 20 dimensions of caregiver burden. Objective burden is divided into six dimensions and is assessed with 25 items. The BIZA-D does not provide an overall load score [66]. Consequently, the BIZA-D is used to document the psychosocial impairments caused by the treatment. The administration time is 45–60 min.

The Subjective Burden Scale (SBS) is a self-rating scale that was composed to assess the subjective burden of caregivers of older people with dementia in Japan. The 14 items of which it is composed not only explore the psychological, emotional, physical, social and financial state of the caregiver but also the quality of life of family members and the relational stress between them. The total score, obtained with the sum of the single scores, ranges from 0 to 56 [67]. The administration time is 20 min. The validity of the scale was investigated in a study conducted by Matsuda in 1999: this study shows that the Subjective Burden scale significantly represents the mental health status of the caregiver and his perspectives regarding the care activity; this suggests good concurrent validity. The usefulness of the scale is also demonstrated by doctors and other professionals to plan, monitor and evaluate the effectiveness of a long-term intervention program. However, the use of the scale outside Japan still requires further studies and insights [68].

The Neuropsychiatric Inventory Distress Scale (NPI-D), developed to evaluate psychopathology in dementia patients, originally evaluated 10 behavioural and psychological symptoms that are common in dementia: delusions, hallucinations, dysphoria, anxiety, agitation or aggression, euphoria, disinhibition, irritability or lability, apathy, and wondering [8]. Two items were introduced (NPI of 12 items): sleep disorders and appetite/eating disorders. The information for the NPI is obtained from a caregiver who knows the patient’s behaviour. Therefore, the total possible NPI score varies from 0 to 144. The “Neuropsychiatric Inventory Caregiver Distress Scale” was developed to evaluate the impact of these 12 symptoms on the family caregiver: a survey scale on the stress perceived by the caregiver was added to the 12 NPI items which is now an integral part of the NPI standard. The score varies from 0 to 60 [69]. The NPI-Q includes both of these additions. Caregivers of dementia patients are to complete the NPI-Q, which is a self-administered questionnaire. NPI-D is a valid and reliable measure widely used to distinguish the frequency and severity of neuropsychiatric changes in dementia as well as caregiver suffering [70]. The administration time is 15 min. The scale allows you to quickly identify the most serious symptom of the disease and, at the same time, the most stressful one, by identifying the most suitable interventions that could reduce the caregiver’s burden, thus improving their quality of life [71].

Due to the variety of scales, mainly divided into home and clinical context, the main objective of the review was to be, in addition to the identification and description of the tools available, a summary useful for identifying the most appropriate tool for a professional healthcare based on the context, patient pathology and speed of administration (as could be Table 1). The further distinction concerns the type of burden that the professional would like to identify: whether subjective, based on the caregiver’s self-perception, or objective, based on items that are able to identify physical, mental, emotional, social and even economic effects. Limitations:

- The review did not consider the individual national, socio-economic and cultural realities in which the instrument was applied.
- A limitation of the research process was to investigate the use of tools without distinction of clinical, intra-hospital and extra-hospital contexts.
- Many scales reported in the literature have been developed mainly for research rather than for clinical purposes and without sufficient information on the clinical context in which to use them.
- None of the scales have been analysed specifically for nursing use but only more generally for use in the clinical setting by various professionals.

## 5. Conclusion

Healthcare professionals can easily use this literature review to understand the scales of burden classification for dementia patients. The scales mentioned can be classified into three categories, with the burden being defined as a one-dimensional, multidimensional, or distinct concept with a subjective and objective component. Studies have shown that the burden assessment of caregivers should include the assessment of both the caregiver and the patient receiving treatment [45]. The NPI-distress scale of Doughtery et al. is a clear example of this, as the patient’s assessment is also based on the caregiver’s burden [45]. In addition to burden classification scales, there have been numerous tools developed in the literature that measure factors like stress, anxiety, and fatigue that arise from nursing activity.

The analysis of these scales showed how the burden of care varies depending on the context of care. The domestic context is the most studied as there is a greater risk of developing the burden of the caregiver. Future research could better study the clinical context.

The socio-cultural context, the employment rate and economic and social factors can also increase or decrease the perception of “care load”. Future research should focus on individual factors and contexts (for example continental or a specific country) and investigate the differences between perceived or subjective burden, and the objective burden related to physical and psychological phenomena.

## Figures and Tables

**Table 1:** Summary table of evaluation Scale of Assessment of Caregiver Care Burden of People with Dementia.

SCALE NAME, AUTHOR, YEAR	ITEMS	SCORE	SAMPLE	STRENGTHS	LIMITATION
**Zarit Burden Interview (ZBI)**, *Zarit, 1980; Zarit & Zarit, 1987. *Short version: **ZBI-18 item **(*Whitlatch, 1991); ***ZBI-12 item **(*Hébert, 2000); ***ZBI-4 item ***(Bédard, 2001)*	22 items version: subjective burden divided into 5 domains: 1) burden in the relationship (6 items), 2) emotional quality of life (7 items), 3) social and family life (4 items), 4) economic aspect (1 item), 5) loss of control over one’s life (4 items).	Score 0–88; ≤20 low or no load, between 21 and 40 light load, between 41 and 60 moderate load, ≥60 high load.	Caregiver of dementia patients.	-Short administration time. -Few items. -Translated into many languages.	-Specific for Parkinson’s disease. -Treats burden only as a one-dimensional variable.
**The Caregiver Burden Inventory (CBI), ** *Novak and Guest, 1989.*	24 items divided into 5 domains: 1) time-dependent burden required by care (items 1–5), 2) developmental burden (items 6–10), 3) physical burden (item 11–14), 4) social burden (items 15–19), 5) emotional burden (items 20–24).	Score 0 (no burden) −100 (maximum perceived burden level.	Caregiver of patients with dementia in the home setting.	-Multidimensional evaluation.	-Patients with Alzheimer’s and dementia. The test may have less reliability due to the emotional and social burden of the caregivers.
**Caregiver Burden scale (CBS), ** *Gerritsen and van der Ende, 1994.*	22 items divided into two sub-scales: 1) Relationship (7 items); 2) personal consequences (6 items) and explore the subjective and objective burden.	5-Point scale (0=strongly disagree, 4=strongly agree). A high score represents a high level of burden.	Home context.	-Measures the burden of care regardless of the diagnosis. -Short administration time.	-The test may have less reliability due to the emotional and social burden of the caregivers.
**Relatives Stress Scale, (RSS), ** *Greene et al., 1982.*	15 items.	5-Point stairs; Score 15–75; a high score indicates a high level of perceived stress.	Caregiver of seniors with dementia.	-Few items. -it can also be used to measure the effectiveness of an intervention and detect the effects on caregivers and patients.	-Average administration time.
**The Neuropsychiatric Inventory Questionnaire Distress scale (NPI-D), ** *Kaufer et al., 1998.*	12 items that investigate the neuropsychiatric symptoms of the patient with dementia.	For the symptoms present, a scale from 0 to 5 must be compiled regarding the level of stress perceived by the caregiver in relation to that symptom (0=not at all and 5 a lot).	Clinic context.	-Short administration time. -Few items.	-Patients with dementia. -The test may have less reliability due to the emotional and social burden of the caregivers.
**Burden Scale for Family Caregivers **(**BSFc) ***Chiu, & Oliver, 2003.*	28 items concerning the perception of subjective burden.	Score 0–84; 0–35 absent or subjective live burden, 36–45 moderate, 46–84 severe to very severe.	Home setting	-Short administration time. -Translated into many languages.	-Many items. -The test may have less reliability due to the emotional and social burden of the caregivers.
**Short form of Burden scale for Family Caregiver (BSFc-s)**, *Gräsel, Berth et al., 2004.*	10 items.	Score 0–30; 0–4 absent or slight subjective burden, 5–15 moderate, 15–30 severe.	Caregiver of subjects with dementia in the home setting.	-Short administration time. -Few items. -Translated into many languages.	-Only home setting. -Patients with dementia and stroke.
**Screen for Caregiver Burden (SCB), ** *Vitaliano, Young, Becker, & Maiuro, 1991.*	25 items concerning 2 domains: subjective and objective burden.	Score for each items 1–4 (1 absence of distress-4 high perceived distress), total score 0 –100. A high score represents a high level of burden.	Home and clinical context.	-Two-dimensional evaluation of the burden.	-Average administration time. -Many items. -Patients with Alzheimer’s disease.
**Burden Assessment scale (BAS), ** *Reinhard, Gubman, Horwitz, & Minsky, 1994.*	19 items: 10 objective burden and 9 subjective burdens.	Each item is evaluated from 0 (never / not at all) to 4 (very frequently) based on the frequency in which the burden is experienced in the various areas of investigation. A high score is linked to a high level of burden.	Home and clinical context.	-Short administration time	-The test may have less reliability due to the emotional and social burden of the caregivers.
**Burden Assessment schedule, ** *Thara, 1998.*	40 items divided into 9 domains: 1) related spouse, 2) physical and mental health), 3) external support, 4) caregiver routines, 5) patient support, 6) accountability, 7) other relationships, 8) patient behaviour, 9) caregiver strategy.	3-point scale; score from 40 to 120 with higher scores indicating a greater care burden.	Home context.	-Multidimensional evaluation of the burden without distinction between subjective and objective.	-People with chronic mental illness. -Many items.
**Berlin inventory of caregivers’ burden with dementia patients (BIZA-D), ** *Zank S, Schacke C, & Leipold B, 2006.*	20 sub-scales with a total of 88 items concerning the subjective and objective burden: 6 sub-scales investigate the assistance and practical support activities and are related to the objective care load (total 25 items), 6 sub-scales investigate the subjective burden of all exposure of behavioural changes and symptoms of the patient (total 26 items), 6 sub-scales concerning the positive aspects and perceived needs in the care activity (total 28 items) and 2 sub-scales concerning the conflicting aspects in the care activity (total 9 items).	A high score is predictive of a high level of objective load (6 sub-scales with 0–16 scoring) and subjective (14 sub-scales with 0–4 scoring).	Home and clinical context.	- Multidimensional evaluation of the burden.	-Patients with dementia -High administration time. -Many items.
**Self-Rated Burden Scale (SRB)**, *van Exel et al.; 2004.*	Scale from 0 to 100 representative of the perceived burden.	Score 0–100; 0=no burden, 100=maximum perceived burden level.	Home and clinical context.	-Short administration time.	-Must be used in combination to complete the load assessment.
**Subjective Burden Scale (SBS), ** *Mastuda, 1999.*	14 items.	4-Point stairs; score 0–56, a high score indicates a high level of subjective burden.	Home context.	-Few items. -It also evaluates the quality of life of family members and the relationship stress between them.	-Elderly patients with dementia. -Average administration time.
**Family Burden Interview Schedule (FBIS), ** *Pai & Kapur, 1981.*	24 items grouped into 6 areas of the burden: 1) economic burden, 2) change and break in routine activities 3) changes and break in family ties 4) changes in leisure time 5) effects on physical health 6) effects on mental health.	3-Point scale: 0 (absence of burden), 1 moderate burden, 2 high burden. Total score 0 – 48, perceived burden.	Home and clinical context.	-Two-dimensional evaluation of the burden.	-Average administration time. -Many items.
**Self-Perceived Pressure from Informal Care Scale (SPPIC), ** *Pot et al., 1995.*	9 items.	Score 0–9 A high score reflects a high level of perceived burden.	Home context.	-Short administration time. -Few items.	-The burden refers to the needs of the assistance situation in proportion to the personal interests of the caregiver, non-care related.
**Montgomery - Borgotta Caregiving Burden Scale ** *Montgomery, 2002, Montgomery et al., 1985; Schene, 1990.*	15 items divided into three domains that investigate: objective burden (6 items), subjective burden (4 items), demand burden (burden of the load) (4 items).	Score 1–5 for each item (5-point scale) For objective burden score 6–30,> 23 high burden level, subjective burden score 4–20,> 13 high burden, for burden demand score 4–20,> 15 high burden.	Home and clinical context.	-Distinguishes between subjective and objective components of the burden and emphasizes the multidimensionality of the impact of treatment.	-Average administration time.
**Caregiver Assessment Difficulties Index (CADI), ** *Nolan & Grant, 1992.*	30 items.	3-point scale: 0=never, 1=sometimes, 2=always. For items with a score of 1 or 2, the perceived burden level must be declared.	Clinical context.	-It can also be used to measure changes in burdens over time.	-Patients with dementia. -Average administration time. -Many items.
**Impact of Alzheimer Disease, ***Cole et al., 2014*.	12 items.	5-Point stairs; score 0–48 with higher scores for high burden level.	Home context.	-Few items.	-Patients with Alzheimer’s.
**Dementia Burden Scale ** **– ** **Caregiver, ** *Peipert et al., 2018.*	34 items corresponding to the sum of the items of three existing scales: NPI-D (12 items), Modified Strain Index Scale (13 items), PHQ-9 (9 items).	Score for each item 0–100; <20 low burden level, a score between 50 and 79 a moderate level, a score> 80 high level.	Home context.	- Multidimensional evaluation of the burden.	-Home setting. -Many items.
